# I223R Mutation in Influenza A(H1N1)pdm09 Neuraminidase Confers Reduced Susceptibility to Oseltamivir and Zanamivir and Enhanced Resistance with H275Y

**DOI:** 10.1371/journal.pone.0037095

**Published:** 2012-08-24

**Authors:** Jérome LeGoff, Dominique Rousset, Georges Abou-Jaoudé, Anne Scemla, Patricia Ribaud, Séverine Mercier-Delarue, Valérie Caro, Vincent Enouf, François Simon, Jean-Michel Molina, Sylvie van der Werf

**Affiliations:** 1 University Paris Diderot, Sorbonne Paris Cité, Paris, France; 2 Assistance Publique-Hôpitaux de Paris, Laboratoire de Microbiologie, Hôpital Saint Louis, Paris, France; 3 Inserm U941, Paris, France; 4 Institut Pasteur, Unité de Génétique Moléculaire des Virus à ARN, Centre National de Référence des virus influenzae (Région-Nord), Département de Virologie, Paris, France; 5 Centre National de la Recherche Scientifique, URA3015, Paris, France; 6 Infectious Disease Department, Hôpital Saint Louis, Paris, France; 7 Service d'Hématologie-Greffe, Hôpital Saint Louis, Paris, France; 8 Institut Pasteur, Plateforme de génotypage des pathogènes et santé publique, Paris, France; University of Edinburgh, United Kingdom

## Abstract

**Background:**

Resistance of pandemic A(H1N1)2009 (H1N1pdm09) virus to neuraminidase inhibitors (NAIs) has remained limited. A new mutation I223R in the neuraminidase (NA) of H1N1pdm09 virus has been reported along with H275Y in immunocompromised patients. The aim of this study was to determine the impact of I223R on oseltamivir and zanamivir susceptibility.

**Methods:**

The NA enzymatic characteristics and susceptibility to NAIs of viruses harbouring the mutations I223R and H275Y alone or in combination were analyzed on viruses produced by reverse genetics and on clinical isolates collected from an immunocompromised patient with sustained influenza H1N1pdm09 virus shedding and treated by oseltamivir (days 0–15) and zanamivir (days 15–25 and 70–80).

**Results:**

Compared with the wild type, the NA of recombinant viruses and clinical isolates with H275Y or I223R mutations had about two-fold reduced affinity for the substrate. The H275Y and I223R isolates showed decreased susceptibility to oseltamivir (246-fold) and oseltamivir and zanamivir (8.9- and 4.9-fold), respectively. Reverse genetics assays confirmed these results and further showed that the double mutation H275Y and I223R conferred enhanced levels of resistance to oseltamivir and zanamivir (6195- and 15.2-fold). In the patient, six days after initiation of oseltamivir therapy, the mutation H275Y conferring oseltamivir resistance and the I223R mutation were detected in the NA. Mutations were detected concomitantly from day 6–69 but molecular cloning did not show any variant harbouring both mutations. Despite cessation of NAI treatment, the mutation I223R persisted along with additional mutations in the NA and the hemagglutinin.

**Conclusions:**

Reduced susceptibility to both oseltamivir and zanamivir was conferred by the I223R mutation which potentiated resistance to both NAIs when associated with the H275Y mutation in the NA. Concomitant emergence of the I223R and H275Y mutations under oseltamivir treatment underlines the importance of close monitoring of treated patients especially those immunocompromised.

## Introduction

Oseltamivir is considered to be the drug of choice for treatment of patients with pandemic influenza, whereas zanamivir is usually restricted to patients with suspected oseltamivir resistant strains.

Until recently, a low frequency of resistance to neuraminidase inhibitors (NAIs) was reported among seasonal as well as A(H5N1) influenza viruses, most often in drug treated and/or immunosuppressed patients [1], [2], [3]. The H275Y substitution in the neuraminidase (NA) of the N1 subtype is the most commonly observed mutation associated with oseltamivir resistance. In H1N1 viruses reported before 2007, it results in low or unstable NA activity, decreased affinity for the substrate, decreased amount of NA on the cell surface, impaired growth in cell culture and decreased viral fitness and transmission [4], [5], [6], [7]. However, natural resistance to oseltamivir in seasonal H1N1 viruses associated with the mutation H275Y in the NA emerged in 2007 in Europe and became predominant worldwide within a year [8], [9]. A permissive genetic background achieved through mutations that pre-empted the H275Y substitution and restored viral fitness of H275Y bearing viruses is likely to account for their widespread diffusion [6], [10], [11], [12]. So far, oseltamivir resistant variants were rarely reported among pandemic A(H1N1) 2009 (H1N1pdm09) influenza viruses: by October 5, 2011, a total of 605 cases have been identified worldwide (18 cases in France) with a high proportion in immunocompromised and/or oseltamivir treated patients [13]. A minority of resistant viruses were detected among patients without known exposure to oseltamivir including one in France [14].

In all cases, resistance was linked to the H275Y mutation which occurred in less than 2% of tested A(H1N1)pdm09 viruses [15] but can reach more than 13% among treated immunocompromised patients [16]. The mutation has been shown to emerge in patients infected with H1N1pdm09 virus as early as 4 days after initiation of oseltamivir treatment and to persist well after cessation of oseltamivir exposure in some immunocompromised patients [16], [17], [18], [19]. The use of zanamivir whatever the route used, inhaled (n = 8), intravenous (n = 5) or nebulised (n = 1), for treatment of patients infected with the H1N1pdm09 virus resistant to oseltamivir has been associated with reduced viral shedding or recovery in most patients (12/14) [17], [18], [20], [21], [22], [23], [24]. Recently, the emergence of an I223R mutation in the NA associated with reduced susceptibility to zanamivir was reported in two immunocompromised and one immunocompetent patients [25], [26], [27]. In immunocompromised patients, this mutation emerged subsequently to or in combination with the H275Y mutation in the NA upon failure of oseltamivir followed by zanamivir treatment.

We report here the selection of the H275Y and I223R mutations in the NA in an immunocompromised patient with sustained H1N1pdm09 virus shedding successively treated by one course of oseltamivir and two courses of zanamivir. Using reverse genetics, we demonstrate that the I223R mutation conferred reduced susceptibility to both NAIs and in the presence of the H275Y mutation potentiated resistance to both NAIs. In this patient, no viruses harboring both mutations were detected. This could be related to the impaired in vitro growth characteristics of the H275Y/I223R double mutant produced by reverse genetics.

## Methods

### Samples

Nasopharyngeal swabs were collected in 3 ml of Universal Transport Medium (UTM) (Copan Diagnostics Inc, Murrieta, CA). Plasma samples collected for surveillance of viral opportunistic infections were used for the detection of H1N1pdm09 RNA in blood and serology.

This study which was not part of a controlled clinical trial, was a non-interventional study with no addition to usual procedures. Biological material and clinical data were obtained only for standard viral diagnostic following physicians' prescriptions (no specific sampling, no modification of the sampling protocol). Data analyses were carried out using an anonymized database. According to the French Health Public Law (CSP Art L 1121-1.1), such protocol does not require approval of an ethics committee and is exempted from informed consent application.

### Virus detection and virus isolation

Total nucleic acid was extracted from 400 µL of UTM or plasma using the EasyMag System (Biomérieux, Marcy l'Etoile, France) and eluted in 100 µl according to the manufacturer's instructions. H1N1pdm09 infection was diagnosed by real-time reverse transcription–PCR (RT-PCR) assay on a 7500 Real Time PCR System (Applied Biosystems, Foster City, CA) according to the protocol designed by the National Influenza Center Northern-France (Institut Pasteur, Paris, France) [28]. Influenza virus isolation was performed in Madin Darby Canine Kidney (MDCK) cells using standard methods. Other viral respiratory infections were investigated by a multiplex molecular assay based on the Multiplex Ligation-dependent Probe-Amplification technology (RespiFinderPlus, Pathofinder, Maastricht, The Netherlands).

### Detection of mutations in the neuraminidase gene by pyrosequencing

For pyrosequencing, viral RNAs extracted from clinical specimens as well as isolates using the NucleoSpin Virus system (Macherey-Nagel, Düren, DE) were amplified with Platinum Taq (Invitrogen, Carlsbad, CA) using pyrosequencing primers (50 µM) specific for each mutation. For the H275Y mutation, primers were as described [29]. For the I223R mutation, primers GRswN1-602Fw (5′- GGGCAGTGGCTGTGTTAAAG-3′) and GRswN1-760Rv-biot (5′-biotine- TGTATGAGGCCTGTCCATCAC-3′) were designed with the Pyrosequencing Assay Design Software (version 1.0; Biotage, Uppsala, Sweden). Pyrosequencing reactions were performed on purified biotinylated amplicons using the PSQ MA 96 platform pyrosequencer (Biotage, Uppsala, Sweden) with Pyrogold reagents according to Biotage recommendations. The sequencing primers (0.40 µM) were H1N1pdm-N1-F804 [29] and GRswN1-642Fw (5′- CACTATCAAGAGTTGGAGA-3′) for detection of the H275Y and I223R mutations, respectively.

### Sequencing of the neuraminidase and hemagglutinin genes

The complete NA and hemagglutinin (HA) coding sequences were determined using Big Dye v.1.1 chemistry on an ABI3730XL capillary sequencer (Applied BioSystems) and were analyzed using BioNumerics software (Applied Maths, Belgium). Accession numbers of Viral isolates are as follows: D0: A/Paris/7976/2010, NA (EPI324954), HA (EPI324955); D6: A/Paris/7975/2010, NA (EPI324951), HA (EPI324952); D11: A/Paris/7154/2009, NA (EPI324942), HA (EPI324943); D19: A/Paris/7974/2010, NA (EPI324948), HA (EPI324949); D25: A/Paris/7973/2010, NA (EPI324945), HA (EPI324946); D45: A/Paris/1156/2010, NA (EPI324957), HA (EPI324958); D69: A/Paris/1157/2010, NA (EPI324960), HA (EPI324961); D83: A/Paris/1158/2010, NA (EPI324963), HA (EPI324964); D98: A/Paris/1159/2010, NA (EPI324966), HA (EPI324967); A/Paris/2590/2009, NA (EPI180570), HA (EPI180568).

### Molecular cloning and sequencing of the region of the NA spanning both 223 and 275 mutations

To characterize quasi species, the NA sequences corresponding to residues 201 to 300 were amplified by RT-PCR with primers 223SLSF (5′-GGGCAGTGGCTGTGTTAAAG-3′) and 223SLSR (5′-ATTCGAGCCATGCCAGTTAT-3′) and cloned using the TOPO TA Cloning kit for sequencing (Invitrogen). Recombinant plasmids were sequenced with 223SLSF and 223SLSR primers using an ABI 3100 Genetic Analyser (Applied Biosystems). Sequences were aligned using Sequence Navigator software version 1.0.1 (Applied Biosystems).

### Construction of recombinant viruses by reverse genetics

An eight-plasmid reverse-genetics system was generated by cloning the cDNAs corresponding to the eight viral segments of the pandemic virus A/Paris/2590/2009(H1N1) at the *Bsm*BI sites in the bidirectional pRF483 plasmid (kindly provided by R. Fouchier, Erasmus MC, Rotterdam, The Netherlands) [30]. Mutations I223R and H275Y in the NA and the N31S mutation in the M2 restoring sensitivity to amantadine were generated using the Quick change II site-directed mutagenesis kit (Stratagene). Recombinant viruses were produced essentially as previously described [31]. The HA, NA and M genes were sequenced using a Big Dye terminator sequencing kit (Applied Biosystems) and an automated sequencer (Perkin Elmer) to verify the presence of the mutations.

### Titration of viruses in plaque assay

Engineered viruses were titrated in a standard plaque assay on MDCK-SIAT1 cells [32]. Briefly, the cells were incubated in the presence of serial dilutions of virus at 35°C for one hour before being overlaid with Avicel-containing medium with 1 µg/ml Tosyl-phenylalanyl-chloromethyl-ketone-treated trypsin. After 72 hours incubation at 35°C, the cells were stained with crystal violet solution containing 10% formamide and plaque numbers determined.

### Replication kinetics assay

The MDCK-SIAT1 cells were incubated with the recombinant virus for 1 hour at 35°C at a multiplicity of infection (MOI) of 0.001. At the end of the incubation period, which was considered the zero time point, the cells were washed twice and fresh medium containing 1 µg/ml TPCK-treated trypsin was added. The supernatants were collected at 12, 24, 36, 48, 60 and 72 hours post infection. To quantify the levels of infectious particles in supernatants, the end-point dilution assay was performed on MDCK-SIAT1 cells using standard procedures [33].The statistical analyses were done using the Graphpad Prism (version 5) software.

### NA enzymatic activity and NA inhibition assays

The enzymatic activity of the NA was measured using the 2′-(4-Methylumbelliferyl)-α-D-N-acetylneuraminic acid (MUNANA) (Sigma) as fluorogenic substrate essentially as described previously [10], [34]. For Km determination, final MUNANA concentration ranging from 10 to 500 µM and for Ki determination, final NAIs ranging from 0.01 nM to 5000 nM were used. Fluorescence was monitored using the TwinkleTM LB970T (Berthold Technologies) fluorimeter.

## Results

### Case Description

On November 20, 2009 (D0), a 24-year-old man was admitted to the infectious disease department with a 10-day old history of fever, coryzal symptoms, diarrhea and cough. The patient had been seen 10 days before admission for a suspicion of catheter infection and had been administrated antibiotic treatment (amoxicillin/clavulanic acid and ciprofloxacin). He was seen two days before admission for follow-up. At this time, a postexposure course of oseltamivir (75 mg×1/d) was started after influenza H1N1pdm09 had been diagnosed for his friend who had similar symptoms. The influenza-like illness syndrome of the patient that would have required a therapeutic dosage of oseltamivir was actually not considered.

The patient's medical history showed type 4 acute myelogeneous leukaemia, diagnosed in January 2008. He had undergone allogeneic cord blood stem cell transplantation in January 2009. Following graft-versus-host complications, the patient had required ongoing immunosuppression with steroids and had presented relapses of leukaemia successively in March and October 2009.

When hospitalized, the patient was being treated with prednisolone (20 mg/d), sunitinib, valaciclovir, amoxicillin, voriconazole, and cotrimoxazole.

A physical examination showed a temperature of 37.2°C. The blood pressure was 130/70 mm Hg, the pulse rate 109 beats per minute, the respiratory rate 15 breaths per minute, and the oxygen saturation 95% on room air (fraction of inspired oxygen 21%). Lung wheezes were audible, and the patient exhibited cough and rhinorrhea. Blood tests showed a neutrophil count of 1.30×10^9^/L, a lymphocyte count of 0.40×10^9^/L, a platelet count of 33×10^9^/L, and a C-reactive protein level of 12 mg/L. Chest radiography showed no abnormalities. The patient was started on amoxicillin-clavulanic acid and ciprofloxacin and oseltamivir was increased to the treatment dose of 75 mg×2/d. A nasopharyngeal specimen collected on D0 tested positive for H1N1pdm09 virus. No other respiratory virus was detected. The patient improved and was discharged on D4, but nasal swabs obtained on D6 and D11 were still positive for H1N1pdm09 virus. While the symptoms did not worsen, but because of the concern for possible oseltamivir resistance, oseltamivir was discontinued and inhaled zanamivir (10 mg twice daily) was started on D15 and discontinued after a total of 10 days. The symptoms did not improve with use of inhaled zanamivir and follow-up nasal swabs taken between D19 and D69 were still positive in the context of persistent neutropenia and lymphopenia. A second 10-day course of zanamivir (10 mg twice daily) was started on D70 due to the persistence of a cough.

The patient was again admitted on D96 with a worsening fever and cough. Oxygen saturation was 95%. Chest radiography and CT-scanner remained unremarkable, but pansinusitis was diagnosed. No bacterial or fungal infections were identified. Broad spectrum antimicrobial therapy (imipenem and voriconazole) was administered without significant improvement. A nasal swab taken on D98 still contained H1N1pdm09 virus but neuraminidase inhibitor therapy was not resumed. No other specimens than nasal washes were collected to assess the presence of H1N1pdm09 throughout the respiratory tract. Blood tests confirmed the relapse of leukaemia with a leukocyte count of 170.00×10^9^/L and 91% of blasts cells. The patient received oral chemotherapy and was discharged home for palliative care on D113. On D139, he was hospitalized after two days of fever and a vomiting episode. After a rapid deterioration of his condition in a context of impaired consciousness, the patient died on D140. The relapse of leukaemia was considered as the cause of death. No autopsy was performed.

### Virus shedding

**Figure 1:**
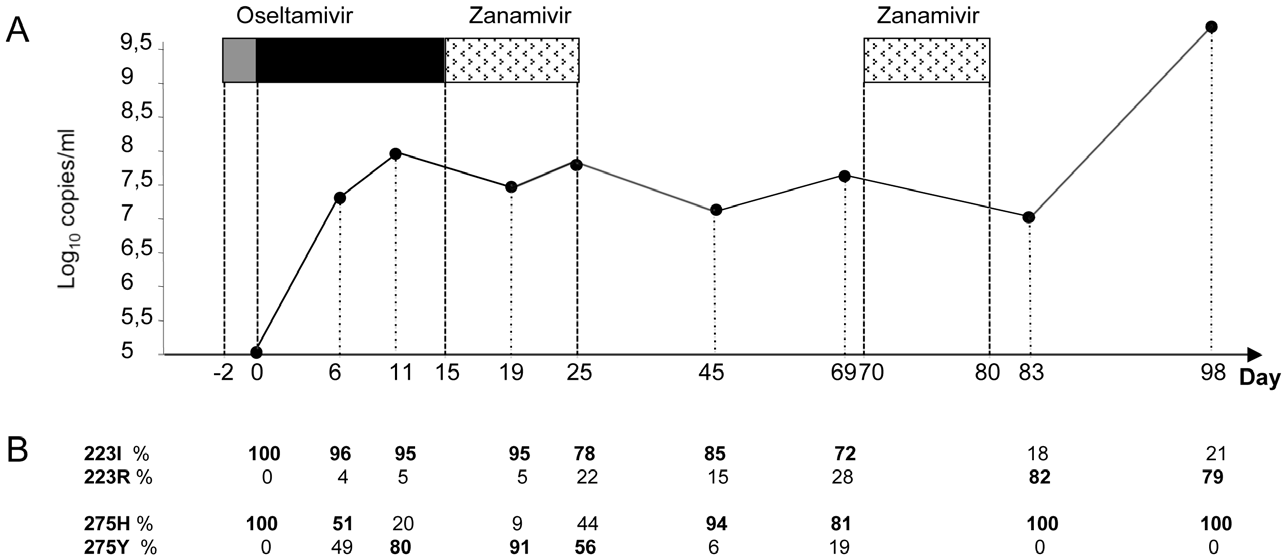
Treatment history and viral evolution. **A**. Evolution of nasal H1N1pdm09 RNA levels and neuraminidase inhibitors treatment history. For each sequential nasopharyngeal sample the level of H1N1pdm09 RNA determined by real-time RT-PCR targeting the M gene is expressed as the log of copies per mL. Neuraminidase inhibitor treatment is represented by boxes: oseltamivir 75 mg×1/d (grey box), oseltamivir 75 mg×2/d (black box), and inhaled zanamivir 10 mg×2/d (stippled box). **B**. Based on the pyrosequencing quantification performed on primary specimens, the proportion of amino acids at positions 223 and 275 in the NA is indicated as a percentage below each sample. The level of the predominant residue is indicated in bold.

During the course of illness, nasal swabs from D0 up to D98 were found positive for H1N1pdm09 virus both by RT-PCR and virus isolation. The viral RNA level as determined by real time RT-PCR showed a slight increase shortly after the start of oseltamivir treatment, with no significant variation between D6 and D83 despite antiviral therapy, and then a significant increase (more than 2 log_10_) 18 days after zanamivir cessation (Fig. 1A). This viral load increase was concomitant with worsening fever and cough and relapse of the leukaemia. In accordance with the immunosuppression in this patient, no significant antibody response was detected by microneutralization (data not shown), which likely contributed to the prolonged viral shedding. To assess if this uncontrolled H1N1pdm09 infection in an immunocompromised host was associated with virus dissemination to the blood we tested for H1N1pdm09 RNA by RT-PCR in plasma samples. In agreement with previous reports, none of the ten plasma samples collected between D0 and D138 were positive for H1N1pdm09 virus.

### Mutations in the neuraminidase and hemagglutinin

Six days after initiation of oseltamivir treatment, the H275Y mutation was detected on both the primary specimen and virus isolate and remained largely predominant (>90%) at least four days after treatment interruption (Fig. 1; Table 1). Despite the absence of selective drug pressure for 54 days the mutation H275Y was still detected on D69 as a minor (19%) population. On day six, a small population of viruses with the I223R mutation in the NA was also observed. During the second course of zanamivir (D70 to D79) the proportion of these I223R mutants increased and persisted at high level (about 80%) at least 19 days after zanamivir cessation (Fig. 1). Rapidly following the detection of the H275Y and I223R mutations in the NA, additional mutations in the HA at positions 374, 415, and 418 were detected (Table 1). Further diversification of the viral sequences was observed after D45 in the HA and the NA. The functional significance of these additional mutations remains unclear at present. However, based on alignment of HA and NA sequences available in databases, mutations at positions 249 and 332 in the NA of H1N1pdm09 viruses have been observed, reflecting natural genetic variations.

**Table 1:** Additional mutations in neuraminidase and hemagglutinin.

			Additional mutations in NA^x^				Additional mutations in HA^x^						
A/California/7/2009	H275	I223	G249	N329	K331	T332	D97	D187	I321	E374	F415	I418	N444
A/NewYork/18/2009									V				
D0^a^													
D6	H/Y	I/R									F/L		
D11	H/Y	I/R								E*-E/K#	F/L		
D19	H/Y	I/R								E*-E/K#	F*-F/L#	I#-L*	
D25	H/Y	I/R								E*-E/K#	F/L	I*-I/L#	
D45	H/Y	I/R					D# -D/N*		I/V	E*-E/K#	F/L	L*- I/L#	
D69	H/Y	I/R	G*-G/E#	N	K*-K/Q#	T*-T/A#			I/V	E*-E/K#	F/L	I*- I/L#	
D83^a^		I/R	G*	D*	K*	T*	D*	D/N*	A/V*	E*	F*	L*	N/K*
D98^b^		I/R	G*-G/E#	N/D	K	T*-T/A#	N*	D/N*	A/V/T/I*	E*	F/L*	L*	N/K*

Only amino acid mutations or mixture of wild type and mutations are indicated.

a HA and NA sequence only from isolate.

b HA sequence only from isolate.

* : detected only on isolates.

# : detected only on clinical specimens.

x : relative to the sequence of A/NewYork/18/2009.

**Figure 2:**
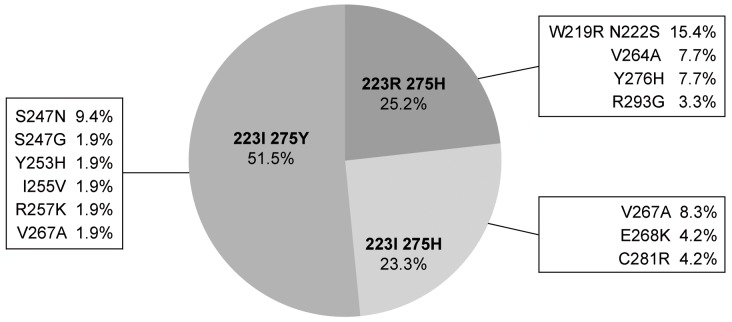
Molecular cloning of the NA gene from the D25 nasal swab. The NA region between residues 201 and 300 was amplified, cloned and sequenced. Frequencies of clones harboring various patterns of amino acids at positions 223 and 275 in the NA are represented. For each pattern, frequencies of clones with additional mutations are indicated.

To determine if the concomitant detection of the I223R and H275Y mutations on the same specimens corresponded to viruses harbouring both mutations in their genome or to a mixture of viruses harbouring either mutation I223R or H275Y, we performed a molecular cloning on the viral RNA extracted from the specimen collected on day 25 when both mutations were readily detected and determined the sequence of the region of the NA spanning both mutations, i.e. residues 201 through 300. Of 103 clones, 53 (51.5%) harbored only 275Y, 26 (25.2%) only 223R and 24 (23.3%) were wild type (223I and 275H) (Fig. 2). None of these clones harbored both 275Y and 223R mutations simultaneously. Interestingly, 18.9% of 275Y, 34.6% of 223R and 16.7% of wild type clones showed various additional mutations. These additional mutations were not found in subsequent sequences, suggesting that they did not confer a significant selective advantage when drug pressure was withdrawn, or during the second course of zanamivir treatment.

### Enzymatic activities of the NA of viral isolates

To evaluate the level of resistance conferred by the mutations detected in the NA that emerged upon treatment, we determined the enzymatic characteristics of the NA of viruses isolated on D0, after treatment with oseltamivir (D19) or zanamivir (D25 and 98) using a functional fluorimetric assay (see methods). The enzymatic parameters of the D0 isolate were in the same range as those of the reference strain A/California/7/2009(H1N1) (Table 2). However, for isolates harboring the H275Y (D19 isolate) or the I223R (D25, D98 isolates) mutations in major proportions (i.e. >60%; Table 2), the Km increased about two-fold indicating reduced affinity for the substrate. The Ki for oseltamivir of the D19 isolate increased 246-fold consistent with the high level of resistance to oseltamivir conferred by the H275Y mutation. For D25 and D98 isolates, in which the H275Y mutation was not detected, the Ki for oseltamivir increased about 8-fold, suggesting that the I223R mutation by itself reduced oseltamivir susceptibility. D0 and D19 isolates showed no significant change in the Ki for zanamivir in agreement with the absence or low (12%) proportion of the I223R mutation. For D25 and D98 isolates harboring the I223R mutation in 64% and 92% of the viral population respectively, the Ki increased more than 4-fold indicating reduced zanamivir susceptibility.

**Table 2:** Enzymatic characteristics of the NA of virus isolates.

Virus	NA aa^a^				Km^b^ +/− SD	Ki OC^b^^,^ c +/− SD	fold	Ki ZAN^b^^,^ d +/− SD	fold
Isolates	223		275		(µM)	(nM)		(nM)	
	I%	R%	H%	Y%					
Cal/7/09	**100**	0	**100**	0	44.35+/−0.25	0.26+/−0.05	na^e^	0.22+/−0.02	na^e^
D 0	**100**	0	**100**	0	59.27+/−8.02	0.28+/−0.07	na^e^	0.34+/−0.01	na^e^
D 19	**88**	12	19	**81**	106.00*+/−1.41	67.97*+/−19.95	246	0.40+/−0.04	1.2
D 25	36	**64**	**100**	0	143.33*+/−11.44	2.34*+/−0.74	8.5	1.45*+/−0.27	4.3
D 98	8	**92**	**100**	0	111.67*+/−4.50	2.47*+/−0.90	8.9	1.65*+/−0.12	4.9

a results of pyrosequencing quantification on isolates obtained after 2 passages on MDCK cells.

b mean±standard deviation of 3 independent determinations.

c Ki for oseltamivir carboxylate.

d Ki for zanamivir.

e na: non applicable.

* significantly different Km or Ki value as compared to the D0 isolate (Student t test. p<0.05).

### Reverse genetics

To further determine the impact of the H275Y and I223R mutations, viruses harboring either or both mutations in the NA were produced by reverse genetics in the context of the A/Paris/2590/2009(H1N1) virus. All mutant viruses were rescued and grew to titers similar to wild-type except for the 275Y/223R mutant for which titers were reduced by 1 log (data not shown). As shown in Table 3, consistent with the enzymatic parameters determined on the patient's isolates, compared to wild-type, the Km increased about two-fold in the presence of either mutation, and more than six-fold for the double mutant. As expected, for the H275Y mutant the Ki for oseltamivir was highly increased (266-fold) with no significant effect on the Ki for zanamivir. For the I223R mutant, the Ki for both oseltamivir and zanamivir (18.4- and 8.1-fold respectively) increased. For the double mutant the Ki for oseltamivir and zanamivir further increased as compared to each single mutant (Ki = 2354.2 vs 101.08 nM, 23.3-fold increase for oseltamivir; Ki = 3.64 vs 1.94 nM, 1.9-fold increase for zanamivir). These results indicate a potentiation of the level of resistance to oseltamivir and to a lesser extent to zanamivir when both mutations are present simultaneously.

**Table 3:** Enzymatic characteristics of the NA of viruses produced by reverse genetics.

	NA aa		Km^a^+/− SD	Ki OC^a^^,^ b+/− SD	fold	Ki ZAN^a^^,^ c+/− SD	fold
RG viruses	223	275	(µM)	(nM)		(nM)	
RG-wt	I	H	45.87±2.64	0.38±0.03	na^d^	0.24±0.02	na^d^
RG-IY	I	Y	74.55*±6.21	101.08*±18.27	266	0.30±0.01	1.3
RG-RH	R	H	113.93*±4.14	7.00*±0.70	18,4	1.94*±0.35	8.1
RG-RY	R	Y	294.78*±1,73	2354.20*±214.68	6195	3.64*±0.60	15.2

a mean±standard deviation of 2 independent determinations.

b Ki for oseltamivir carboxylate.

c Ki for zanamivir.

d na: non applicable.

* significantly different Km or Ki value as compared to RG wild type (Student t test. p<0.05).

**Figure 3:**
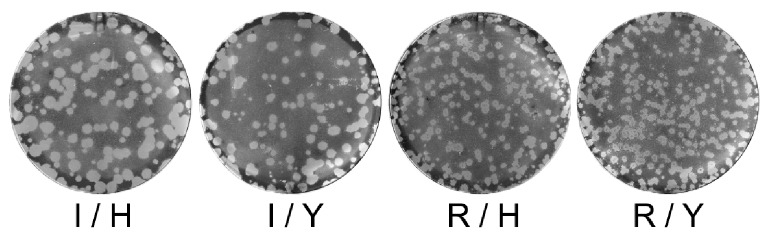
Growth of recombinant viruses with H275Y and/or I223R mutations in MDCK-SIAT1. Viruses produced by reverse genetics derived from the A/Paris/2590/2009(H1N1) strain and harboring the H275Y and I223R mutations alone or in combination as indicated were titrated on MDCK-SIAT-1 cells in a standard plaque assay [31]. Plaque diameters from 398 plaques (86. 87. 126. 99 plaques for RG-wt (I/H). RG-I/Y. RG-R/H and RG-R/Y. respectively) were measured.

Given the impact of the mutations on the Km which indicates a reduced affinity of the NA for its substrate, we examined the virus growth on MDCK-SIAT1 cells in a standard plaque assay and measured the plaque diameters. Mean diameters ± SD were as follows: wild-type (I/H). d = 17.23±3.65; I/Y. d = 14.32±2.45; R/H. d = 8.94±2.47 and R/Y. d = 8.52±1.34. Differences were found to be significant for RG-R/H vs RG-wt(I/H), RG-R/Y vs RG-wt(I/H), RG-I/Y vs RG-R/H and RG-R/Y vs RG-I/Y in a Student t-test (p<0.0001). In addition, the virus growth kinetics were analyzed on MDCK-SIAT1 cells (Fig. 3).

**Figure 4:**
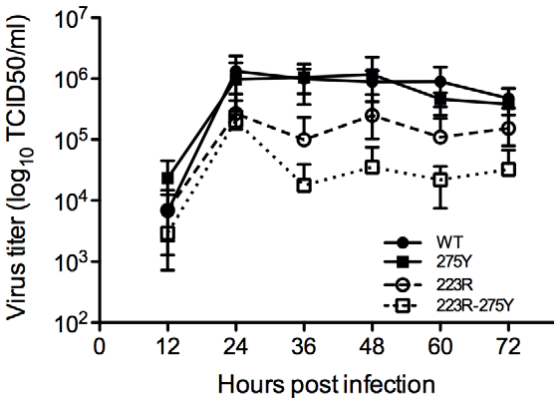
Replication kinetics of recombinant viruses in MDCK-SIAT1. MDCK-SIAT1 cells were inoculated at a multiplicity of infection (MOI) of 0.001 PFU with the engineered viruses. Supernatants were harvested after at 12, 24, 36, 48, 60 and 72 hours post infection and were titrated on MDCK-SIAT1 cells. The results shown are from two independent experiments. Mean titers and standard deviations are shown.

Virus growth was slightly impaired by the H275Y mutation and even more by the I223R mutation. A cumulative effect was seen for the double mutant virus, which formed very small plaques and replicated to lower titers suggesting reduced viral fitness when both mutations were present simultaneously (Fig. 3 and 4).

## Discussion

As no vaccine was available when the influenza A(H1N1) 2009 pandemic started, NAIs, particularly oseltamivir, were the first-line strategy to combat the virus. Oseltamivir has thus been used as primary pharmacologic intervention for managing ill patients or their contacts. We showed in a severely immunocompromised patient, the selection of the H275Y mutation in the NA of the H1N1pdm09 virus 6 days after the initiation of oseltamivir treatment that was preceded by two days of oseltamivir chemoprophylaxis. While the appropriate oseltamivir dose for chemoprophylaxis in immunocompromised hosts is uncertain, the use of prophylaxis despite evidence of upper respiratory tract infection was probably a trigger for oseltamivir resistance selection. The H275Y mutation induced high-level resistance to oseltamivir which was consistent with previous reports [5], [10]. This suggests that oseltamivir monotherapy is likely not effective enough to control influenza shedding in severely immunocompromised patients and may account for resistance selection. Despite no selective drug pressure, the 275Y mutation remained detectable for 54 days.

Although H1N1pdm09 H275Y mutants remain susceptible to zanamivir, the use of this drug had no impact on viral shedding in this patient. Until recently, there have been only few reports of significant zanamivir resistance associated with mutations in the NA gene amongst human influenza viruses, in part due to a more limited use of this molecule: R152K for B virus in an immunocompromised patient [35] and Q136K/L and R371K for A or B viruses in tissue culture and *in vivo* [36], [37], [38]. For the H1N1pdm09 virus, two reports have shown the emergence of an I223R mutation in the NA subsequently in two immunocompromised patients upon failure of treatment with NAIs [25], [26]. In one case, the patient received oseltamivir and then intravenous zanamivir therapy following the emergence of the H275Y mutation that conferred resistance to oseltamivir [26]. The I223R mutation was detected upon relapse of virus excretion after discontinuation of zanamivir therapy at a time when the H275Y mutation could no longer be detected and persisted during the second course of zanamivir therapy. In the second case, the I223R mutation was detected along with the H275Y mutation following oseltamivir therapy and prior to the initiation of intravenous zanamivir therapy [25]. It is not known whether both mutations were simultaneously present on the same virus and the fate of the mutations after cessation of oseltamivir therapy and during zanamivir treatment was not documented. In the two cases, the occurrence of I223R was associated with an increase of the oseltamivir and zanamivir 50% inhibitory concentrations, relative to wild-type and H275Y mutant viruses, respectively.

In the case reported here, during oseltamivir treatment and before the initiation of zanamivir therapy, the I223R mutation in the NA, was detected in a minor population and became predominant when zanamivir was given. Substitutions of residue I223 for V, T or M were observed among seasonal influenza A and B or A(H5N1) viruses under oseltamivir pressure especially in immunocompromised patients [39] or after passages in vitro [40], [41]. Alone these three substitutions caused a minor decrease in oseltamivir susceptibility with no impact on zanamivir susceptibility but in combination either with H275Y or E119V they conferred major resistance to oseltamivir. In H5N1 viruses, under oseltamivir pressure, an I223M substitution in the NA was selected *in vitro* in combination with the H275Y mutation [41]. This dual mutation had a greater impact on the resistance to oseltamivir than H275Y alone but did not confer resistance to zanamivir and showed reduced susceptibility to peramivir. An I223V substitution was detected in the NA of H1N1pdm09 isolates in two summer campers who had received oseltamivir chemoprophylaxis and presented also with the H275Y mutation [42]. Engineered viruses harbouring the mutation I223V in their NA showed a decreased sensitivity to oseltamivir and peramivir and enhanced resistance to both NAIs in the presence of the mutation H275Y [43]. An I223K mutation was also reported to reduce susceptibility to NAIs [25]. These limited data suggest that amino acid substitutions occurring at position 223 may be selected for by oseltamivir along with the H275Y mutation. Here, the association of the I223R mutation with the reduction in zanamivir susceptibility (5 to 7-fold) was demonstrated *in vitro* on viral isolates and viruses produced by reverse genetics. In addition the I223R mutation conferred reduced susceptibility to oseltamivir (9 to 18-fold). These results suggest that the I223R substitution was selected for at low levels under oseltamivir treatment and eventually became predominant after zanamivir treatment. However, the modest level of resistance to zanamivir cannot explain *per se* the treatment failure considering that the local concentrations of zanamivir are expected to be more than 337- and 135-fold higher than the IC50s for influenza virus NAs respectively in nasal mucosa and pulmonary epithelium after a 10 mg dose given twice daily [44]. The relapse of leukaemia with profound immunodeficiency and absence of antibody response is also likely to account for lack of viral control.

In contrast to the H275Y mutation, the I223R mutation persisted at least 19 days after the last zanamivir inhalation but its disappearance thereafter cannot be excluded as viral follow-up was ceased. Potentially reduced viral fitness of 223R viruses was suggested by the reduction in plaque diameter and in virus titers achieved upon growth on MDCK-SIAT1 cells in agreement with a recent report [45] but in contrast to another study which showed that 223R viruses grew to higher titers on ST6GalI-MDCK cells [46]. In the patient studied here, persistence of 223R viruses was associated with a rebound of influenza virus shedding concomitantly with worsening fever, cough and pansinusitis. Upon prolonged virus shedding, emergence of additional mutations which persisted in the HA and in the NA was observed indicating that the viruses evolved with increasing viral population complexity. These additional mutations did not confer a higher level of resistance to zanamivir. Whether they actually improved viral fitness, and thus enabled the persistence of the I223R mutation in H1N1pdm09 viruses deserves further analysis. Viruses harboring both I223R and H275Y mutations produced by reverse genetics showed increased resistance to both zanamivir and oseltamivir compared to single mutants as also shown by Pizzorno et al. [46]. Furthermore, the simultaneous presence of both mutations resulted in further reduced plaque diameter and reduced virus growth on MDCK–SIAT1 cells, as compared to single mutants suggesting that the fitness of the double mutants was significantly impaired. This was in contrast to the observations made by Pizzorno et al. which showed similar growth for wild-type and double mutant viruses on ST6GalI-MDCK cells [46]. However, the likely reduced fitness for the viruses harboring both I223R and H275Y mutations is supported by the fact that double mutants were not detected in the clinical sample positive for both mutations.

This study clearly demonstrates that the mutation I223R alone confers reduced susceptibility to both oseltamivir and zanamivir and was primarily selected by oseltamivir. Despite zanamivir cessation, I223R mutants persisted along with additional mutations in the NA and HA. This highlights the potential for evolution of H1N1pdm09 viruses resistant to NAIs and the risk for possible emergence of sufficiently fit resistant strains able to widely spread. As prophylactic use (1×75 mg per day) has been associated with the selection of the H275Y mutation and potentially could be involved in the selection of the I223R mutation that confers resistance to both oseltamivir and zanamivir, the therapeutic dose of oseltamivir (2×75 mg per day) for prophylaxis should be carefully considered especially in immunocompromised hosts. Restricting the use of neuraminidase inhibitors only to treatment of patients that have ILI symptoms could also be an option to reduce the potential of generating resistance.

Because immunocompromised patients are at risk for sustained influenza virus shedding and for resistance selection, close monitoring and reporting of resistance to NAIs are essential. To limit the spread of any new resistant strain from this population, the promotion of prophylactic measures including influenza vaccination of relatives and healthcare providers should be encouraged as well as reinforcement of infection control practices. This study along with other reports of an I223R mutation reminds the need to develop new drugs with different viral targets.
